# The Bedding and Linen Department

**Published:** 1908-05-09

**Authors:** 


### Nursing Administration.

### THE BEDDING AND LINEN DEPARTMENT.

### 2.—THE LADIES' LINEN LEAGUE.

There are numerous hospitals both in town and country where the cost of the linen department presses very heavily on an overburdened exchequer. Here is the cost of bedding and linen in certain representative hospitals during the year 1906:—

| | £ | | £ |
|---|---|---|---|
| The London Hospital | 1,583 | Birmingham Queen's ... | 142 |
| St. Thomas's... ... | 1,039 | Radcliffe Infirmary ... | 173 |
| Guy's ... ... ... | 742 | Edinburgh Royal ... | 394 |
| The Middlesex ... | 309 | Leicester Infirmary ... | 231 |
| Charing Cross ... | 35 | Royal Hants ... ... | 125 |
| King's College ... | 484 | Royal Berks ... ... | 66 |
| The Royal Free ... | 146 | Bradford Infirmary ... | 186 |
| The Metropolitan ... | 170 | York County ... ... | 112 |
| Birmingham General | 54 | Royal Portsmouth ... | 142 |

It is one of those departments which, when generously maintained, reflects great credit on an institution. Unfortunately it is not always easy to persuade those who have the disbursements in their hands that this is the case. The lay governors of a hospital sometimes fail to perceive the necessity for those enormous supplies on which nurses and doctors must depend to fulfil their duties towards the patients. To appreciate the value of plenty of clean linen a woman's mind is needed; and on this theory the success of the Ladies' Linen League depends. The object of the League is to induce ladies residing within the sphere of the hospital to help in replenishing the household linen. The president is usually some lady of position in the neighbourhood, whose support acts as a guarantee to those whom it is hoped to interest that the Guild or League is founded on the right lines and worthy of support. It is her part to invite vice-presidents to co-operate with her for the purposes of the League. To each vice-president a district is assigned, within which it is her duty to find helpers willing to become associates, and to receive their work and subscriptions annually. The vice-president often holds working parties, at which the work is put in hand, patterns are distributed, and the interest of the members is quickened. The associates agree to subscribe a small sum annually, the minimum being usually 1s. 6d. or 2s. 6d., and to contribute two articles annually from a list which is prepared by the matron. Where workers can be enlisted whose means do not permit of their contributing both money and work one or other is to be deemed a sufficient qualification for becoming an associate. The rules stipulate for the exact following of the measurements supplied, and for the articles, together with subscriptions, being sent in to the vice-presidents in good time before the end of each year. The work is on view on the occasion of an "At Home," when the hospital is thrown open for inspection by the associates and their friends. By this means new interest is kindled in the work of the hospital, and many fresh workers are often enrolled. One of the earliest institutions to found an association of this description was the Lincoln County Hospital, and we quote the list of the articles which the associates are invited to send in with prices and measurements:—

DETAILS OF THE LINEN REQUIRED AT THE COUNTY HOSPITAL.

Prices and Measurements.

100 sheets (linen), length 3¼ yds., width 2 yds., 2s. 3d. per yd.

100 sheets (cotton), length 3¼ yds., width 2 yds., 1s. 6d. to 1s. 9d. per yd.

100 draw sheets (linen), length 3 yds., width 2 yds., 1s. 6d. per yd.

100 draw sheets (cotton), length 3 yds., width 2 yds., 1s. per yd.

100 cot sheets, length 2 yds., width 1½ yds., 1s. 2d. to 1s. 4d. per yd.

100 cot draw sheets, length 1½ yds., width 1½ yds., 1s. per yd.

100 pillow-cases (linen) with tapes, *not* buttons, length 35 ins., width 20 ins.

100 pillow-cases (cotton) with tapes, *not* buttons, length 35 ins., width 20 ins.

100 towels (not rough), length 1½ yds., 1s. 2d. per yd.

100 towels (not rough), length 1 yd., 10½d. per yd.

Counterpanes (white), 7s. 9d. each.

Table-cloths, length from 3 yds. to 4 yds.

Blankets (white), 12s. 9d. per pair.

Cot blankets (white), 6s. 3d. per pair.

*The above prices are net, and include the discount which is allowed.*

The names of local firms who have consented to grant discount for the hospital supplies is appended, and those who contribute the articles can not only choose exactly what they will make, but can buy the material themselves. Many people who would hesitate a long time before giving a subscription of even five shillings in hard cash are found to take a real pleasure in adding to their household purchases a pair of sheets for the hospital, and the reflection that their labours have made some poor sufferer comfortable, to whom the touch of clean linen has perhaps been a luxury long untasted.

Wherever an organisation of this description has been started the result has been to lift the burden of the linen department entirely off the treasurer's hands, and to rejoice the matron's heart with abundance of material to stock her wards. The ultimate success often outstrips the immediate aims. When the list is exhausted it is easy to add bed jackets for the patients' use, and warm winter garments for those leaving in destitution. The money contributed is frequently sufficient to provide not only blankets but nursing requisites, such as air-pillows, water-beds, mackintoshes, and other comforts for the patients, and where influential committees are formed through the efforts of the vice-presidents, the League may even be enabled to support a special bed or cot by their exertions. The effect is to spread an interest in the hospital in quarters where few would look for support, and by drawing on personal service the interest is rendered living and permanent. In every locality, however poor, the invitation to contribute the work of their hands would, we believe, be warmly responded to by women of every class, and the spur to private enthusiasm is not the least important result.

